# Application of Digital Polymerase Chain Reaction (dPCR) in Non-Invasive Prenatal Testing (NIPT)

**DOI:** 10.3390/biom15030360

**Published:** 2025-03-01

**Authors:** Ying Guo, Pimlak Charoenkwan, Kuntharee Traisrisilp, Wirawit Piyamongkol, Fuanglada Tongprasert

**Affiliations:** 1 Department of Obstetrics and Gynaecology, Faculty of Medicine, Chiang Mai University, Chiang Mai 50200, Thailand; ying_guo@cmu.ac.th (Y.G.); kuntharee.t@cmu.ac.th (K.T.); wirawit.p@cmu.ac.th (W.P.); 2 Department of Obstetrics and Gynecology, The First Affiliated Hospital of Dali University, Dali 671000, China; 3 Department of Pediatrics, Faculty of Medicine, Chiang Mai University, Chiang Mai 50200, Thailand; pimlak.c@cmu.ac.th; 4 Thalassemia and Hematology Center, Faculty of Medicine, Chiang Mai University, Chiang Mai 50200, Thailand

**Keywords:** digital PCR (dPCR), non-invasive prenatal testing (NIPT), chromosomal aneuploidy, chromosomal microdeletions and microduplications, monogenic disease

## Abstract

This article reviews the current applications of the digital polymerase chain reaction (dPCR) in non-invasive prenatal testing (NIPT) and explores its potential to complement or surpass the capabilities of Next-Generation Sequencing (NGS) in prenatal testing. The growing incidence of genetic disorders in maternal–fetal medicine has intensified the demand for precise and accessible NIPT options, which aim to minimize the need for invasive prenatal diagnostic procedures. Cell-free fetal DNA (cffDNA), the core analyte in NIPT, is influenced by numerous factors such as maternal DNA contamination, placental health, and fragment degradation. dPCR, with its inherent precision and ability to detect low-abundance targets, demonstrates robustness against these interferences. Although NGS remains the gold standard due to its comprehensive diagnostic capabilities, its high costs limit widespread use, particularly in resource-limited settings. In contrast, dPCR provides comparable accuracy with lower complexity and expense, making it a promising alternative for prenatal testing.

## 1. Introduction

Prenatal testing has undergone a remarkable transformation with the advent of non-invasive prenatal testing (NIPT). The discovery of cell-free fetal DNA (cffDNA) in maternal plasma by Lo YM et al. in 1997 was a breakthrough in prenatal screening, revealing the presence of short, low-concentration fetal DNA fragments that could be analyzed using maternal blood samples [1]. This finding demonstrated the feasibility of using maternal blood for the non-invasive analysis of fetal genetic material, significantly reducing the need for invasive procedures like amniocentesis and chorionic villus sampling (CVS), which carry a small but notable risk of miscarriage [2]. Early studies primarily utilized PCR-based methods for cffDNA analysis, focusing on targeted applications such as fetal sex determination and RhD blood group testing [3,4]. Although these methods were limited to single-gene or locus-specific conditions, they laid the groundwork for the application of advanced technologies like Next-Generation Sequencing (NGS) and digital polymerase chain reaction (dPCR) in cffDNA detection.

The introduction of NGS revolutionized NIPT by enabling the high-throughput sequencing of cffDNA. In 2008, Stephen Quake’s team at Stanford University proved the feasibility of whole-genome shotgun sequencing, an approach based on massively parallel sequencing (MPS) underpinned by NGS technology, for detecting fetal chromosomal abnormalities using maternal plasma. This marked a pivotal moment in the early application of MPS in NIPT [5]. In 2011, Lo YM’s team in Hong Kong published a clinical validation study, establishing the accuracy and reliability of MPS for trisomy 21 detection [6]. That same year, in August, NIPT was introduced in Hong Kong for clinical use as the NIFTY (Non-Invasive Fetal Trisomy) test [7]. Soon after, in October, the commercial launch of MaterniT21^®^ in the USA represented a transformative moment in the global adoption of NIPT [8,9]. Over time, its applications have expanded to cover microdeletions, duplications, and certain single-gene disorders, establishing NGS-based NIPT as an increasingly integrative approach to genetic testing [10]. However, challenges such as high costs, complex infrastructure requirements, and technical limitations in analyzing samples with low fetal DNA fractions—especially in early pregnancy or cases involving maternally inherited mutations—underscore the need for complementary technologies [11].

dPCR has become a valuable tool in molecular diagnostics, with applications spanning oncology, infectious diseases, and NIPT. The concept of dPCR was first introduced in 1992, with the term “digital PCR” formalized in 1999 [12,13,14,15]. Its commercial development began in 2006/2007 with the launch of systems based on microfluidic chips and microarrays, marking its initial transition into broader research and clinical use [16]. The introduction of droplet-based dPCR (ddPCR) systems in 2011, which utilized water–oil emulsions to create thousands of microreactors, dramatically improving precision and throughput [13]. Further advancements, including nanoplate-based platforms in 2020, expanded the range and efficiency of dPCR for clinical applications, particularly in prenatal diagnostics [17]. In the context of NIPT, dPCR addresses key limitations of NGS, such as its sensitivity to low fetal DNA fractions and maternal DNA interference, while also offering superior resistance to PCR inhibitors [18,19]. By the 2020s, dPCR was successfully applied to detect chromosomal aneuploidies, monogenic disorders, and chromosomal microdeletions/duplications, addressing clinical needs requiring exceptional accuracy [20]. Its lower cost and streamlined workflows make dPCR a complementary technology to NGS in scenarios requiring targeted and cost-effective analyses [21].

This review provides an overview of the advancements in dPCR for NIPT, highlighting its potential to address unmet clinical needs in prenatal diagnostics. It explores how the unique characteristics of cffDNA pose technical challenges and influence the design of NIPT technologies. Additionally, this review evaluates the strengths and limitations of dPCR, showcasing its precision and affordability as significant advantages while acknowledging its narrower detection scope and lower throughput compared to NGS. dPCR continues to evolve, addressing key challenges in NIPT while solidifying its role as a critical complement to existing prenatal diagnostic technologies.

## 2. The Role of cffDNA in NIPT: Biological Insights, Applications, and Technological Comparisons

The unique characteristics of cffDNA have established it as an essential biomarker for NIPT, supported by highly sensitive detection technologies such as dPCR, NGS, and quantitative PCR (qPCR) [21,22]. Its distinct fragmentation patterns and molecular stability set cffDNA apart from maternal cell-free DNA (cfDNA), making these differences crucial for its effective application in NIPT.

### 2.1. Fragmentation Characteristics and Molecular Stability of cffDNA

**Figure 1:**
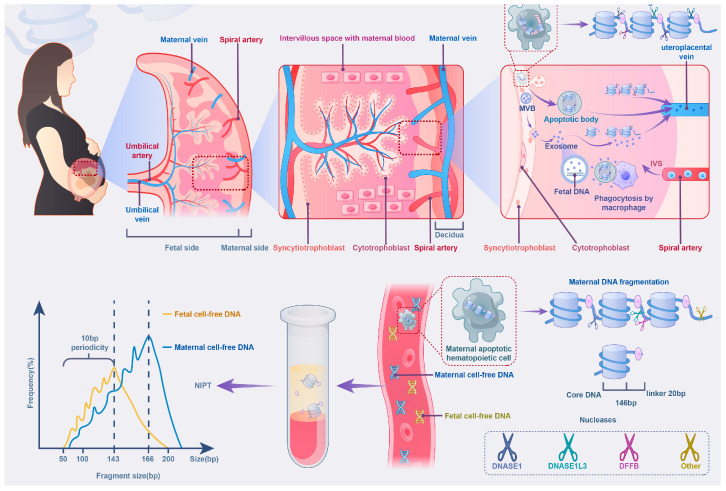
Illustration of the origin, fragmentation, and size distribution of cffDNA and maternal cfDNA. CffDNA is primarily released from apoptotic syncytiotrophoblast cells in the placenta and is also enclosed within exosomes, a type of extracellular vesicle that may protect DNA from enzymatic degradation. Nucleases like DNASE1 and DNASE1L3 cleave DNA at nucleosome cores, generating shorter fragments with a 10 bp periodicity. Maternal cfDNA, derived from apoptotic hematopoietic cells, is cleaved in linker regions between nucleosomes, producing longer fragments. These fragmentation patterns are reflected in the size distribution graph, where cffDNA peaks at approximately 143 bp (yellow line) and maternal cfDNA at 166 bp (blue line), emphasizing their distinct profiles. Abbreviations: NIPT, non-invasive prenatal testing; DNASE1, Deoxyribonuclease 1; DNASE1L3, Deoxyribonuclease 1 Like 3; DFFB, DNA Fragmentation Factor Subunit Beta; MVB, Multivesicular Body; IVS, intervillous space; bp, base pairs. The illustration is based on findings from previous studies [23,24,25,26,27,28,29,30].

CffDNA fragments are typically shorter than maternal cfDNA fragments, with fetal DNA peaking at around 143 base pairs (bp) and maternal DNA peaking at 166 bp [23]. This size disparity arises from their different biological origins and cleavage patterns (Figure 1).

CffDNA is primarily released through the apoptosis of placental trophoblast cells, generating hypomethylated DNA fragments that are highly susceptible to enzymatic cleavage by nucleases such as DNASE1 and DNASE1L3. This enzymatic activity primarily targets nucleosome cores, resulting in shorter fragments with a characteristic 10 bp periodicity, reflecting the organized packaging of cffDNA around nucleosomes [24,25,26,27,28]. In addition to apoptotic release, cffDNA may also be transported via placental exosomes (pEXOs) into maternal circulation. pEXOs are secreted by syncytiotrophoblasts into the intervillous space, from where they enter maternal blood and interact with circulating immune cells, particularly monocytes [29]. Unlike apoptotic release, where cffDNA is freely exposed in circulation, exosome-associated cffDNA is enclosed within vesicles, which may offer protection from enzymatic degradation and influence its stability. However, the relative contribution of pEXOs to the total fetal DNA pool and whether pEXO-derived cffDNA exhibits distinct fragmentation characteristics remain unclear [30]. Further research into the interplay between apoptotic and exosomal pathways could refine NIPT strategies and enhance detection accuracy.

In contrast, maternal cfDNA, predominantly derived from hematopoietic cells, is cleaved between nucleosomes, within linker regions, leading to longer fragments. The unique molecular properties of cffDNA, including its shorter fragment length, rapid enzymatic turnover, and hypomethylation status, form the basis for its effective use in NIPT. These characteristics enable the differentiation of fetal DNA from maternal cfDNA and guide the development of advanced methodologies for prenatal diagnostics. Both NGS and dPCR leverage these properties but through distinct approaches tailored to their technical strengths and clinical applications.

#### 2.1.1. Preanalytical Factors Affecting cffDNA Quality and Detection

The reliability of NIPT depends not only on the biological characteristics of cffDNA but also on preanalytical factors affecting its yield, purity, and stability. The choice of anticoagulants in blood collection tubes significantly impacts cffDNA integrity. Standard EDTA tubes are commonly used, requiring sample processing within 2 h at room temperature or within 6 h at 4 °C to minimize maternal cfDNA contamination. Delayed processing causes maternal white blood cell lysis, releasing additional cfDNA into plasma and lowering the fetal fraction (FF) [31]. To mitigate this, cell-stabilizing tubes (e.g., Streck tubes) contain preservatives that prevent leukocyte lysis and maintain cffDNA stability for up to 7 days at room temperature, reducing preanalytical variability [32].

Proper plasma separation is essential to minimize maternal cfDNA interference. A two-step centrifugation process is commonly used: an initial low-speed centrifugation (e.g., 1600× *g*, 10 min) to separate plasma from blood cells, followed by high-speed centrifugation (e.g., 16,000× *g*, 10 min) to remove residual cellular debris. Improper or delayed processing increases maternal cfDNA contamination, which compromises NIPT sensitivity by reducing the FF [33,34].

After plasma separation, temperature and storage conditions influence cffDNA stability. While immediate freezing at −80 °C is ideal for long-term preservation, prolonged storage at 4 °C or −20 °C may lead to gradual degradation due to nuclease activity, emphasizing the need for proper storage conditions [31].

FF is a key determinant of NIPT accuracy, with levels below 4% increasing the risk of test failure and reducing sensitivity for detecting fetal genetic abnormalities. Standardized preanalytical procedures, including timely sample processing, optimized centrifugation, and appropriate storage methods, are critical for ensuring reliable cffDNA detection in clinical applications [35].

#### 2.1.2. Comparison of NGS and dPCR in Utilizing cffDNA Characteristics

NGS employs genome-wide or targeted methylation sequencing approaches, such as whole-genome bisulfite sequencing (WGBS) or reduced representation bisulfite sequencing (RRBS), to comprehensively profile methylation patterns [36,37]. These methods enable the detailed detection of fetal DNA by distinguishing hypomethylated fetal DNA from highly methylated maternal cfDNA and facilitate the identification of genetic abnormalities. Conversely, dPCR targets specific methylation sites, such as regions rich in cytosine–phosphate–guanine (CpG) dinucleotides in Ras Association Domain Family Member 1, isoform A *(RASSF1A)*, using methylation-specific primers and probes [38]. This targeted approach achieves high sensitivity and specificity for fetal DNA detection, offering faster and more cost-effective solutions for specific clinical applications.

With a half-life of 30 min to an hour, cffDNA analyzed by NIPT reflects the current pregnancy, minimizing interference from the residual cfDNA of prior pregnancies. This rapid turnover, which may vary with gestational age and maternal–fetal conditions, highlights its pregnancy-specific characteristics [39,40]. Both NGS and dPCR leverage this property. However, the time-consuming workflows of NGS may limit its ability to capture real-time changes compared to dPCR. By directly quantifying fetal DNA, dPCR is better suited for dynamic monitoring and timely clinical applications.

The shorter fragment length of cffDNA, peaking at around 143 bp, is leveraged by size-selection methods like gel electrophoresis and microfluidic systems to enrich fetal DNA and reduce maternal cfDNA interference [41,42]. Both NGS and dPCR utilize this property but differ in their approaches. dPCR targets shorter DNA fragments, with amplicon lengths typically ranging from 60 to 150 bp, enabling efficient amplification and high sensitivity even in samples with low fetal DNA fractions. In contrast, NGS relies on high-throughput sequencing for comprehensive genomic analysis, making the two technologies complementary in their applications.

### 2.2. Placental Heterogeneity and the Regulation of cffDNA Release

Placental heterogeneity, which refers to the structural and functional variability within different regions of the placenta, plays a critical role in regulating the release of cffDNA into maternal circulation [43]. This variability affects both the efficiency and pattern of cffDNA release, primarily due to differences in factors such as regional blood flow, oxygen supply, and overall cellular health. Syncytiotrophoblasts, the main source of cffDNA, undergo apoptosis to release fetal DNA fragments, and variations in placental health can lead to differences in the rates of cffDNA release and fragment size. These factors ultimately impact the quantity and quality of fetal DNA available for detection through NIPT, complicating the interpretation of results in some cases [44].

#### 2.2.1. Trophoblast Apoptosis and Oxygenation Impact on cffDNA Release

A key aspect of how placental heterogeneity regulates cffDNA release lies in the role of trophoblast apoptosis and oxygenation. In regions with adequate blood perfusion and oxygenation, the controlled apoptosis of syncytiotrophoblasts ensures a steady and regulated release of cffDNA. Conversely, in regions affected by hypoxia or reduced blood flow, such as those seen in conditions like preeclampsia (PE) and fetal growth restriction (FGR), cellular stress can lead to premature and intensified apoptosis. This pathological process produces fragmented and degraded cffDNA, with hypoxic stress leading to shorter and less stable DNA fragments. Such variability in apoptosis and oxygenation directly affects the size, stability, and total quantity of cffDNA in maternal plasma, posing challenges for the reliability of NIPT, especially in pathological pregnancies [45,46].

Both structural and genetic heterogeneity within the placenta contribute to challenges in interpreting cffDNA-based NIPT results. Genetic differences across placental regions, as seen in confined placental mosaicism (CPM), add another layer of complexity in diagnosing fetal genetic abnormalities.

#### 2.2.2. Role of Confined Placental Mosaicism (CPM)

CPM refers to the presence of genetically distinct regions within the placenta that may not mirror the genetic profile of the fetus. In cases of CPM, placental regions with abnormal genetic material, such as trisomies, often undergo increased apoptotic activity. This elevated apoptosis results in a disproportionate release of cffDNA from these abnormal regions into the maternal plasma. As a result, the cffDNA composition in maternal plasma may misrepresent the true genetic status of the fetus, complicating the interpretation of NIPT results. For instance, a trisomic placental region might contribute an excess of abnormal cffDNA, potentially leading to a false-positive result when the fetus is unaffected. Conversely, normal placental regions may dilute abnormal signals, increasing the risk of a false-negative outcome [47]. Understanding how CPM affects the distribution and release of cffDNA is crucial for accurately interpreting prenatal test results and mitigating the risks of false-positive or false-negative outcomes.

CPM is not uncommon, with studies suggesting it occurs in about 1–2% of pregnancies, particularly in high-risk cases [48,49,50]. In these situations, CPM can significantly impact the accuracy of NIPT, leading to diagnostic challenges. It is crucial to consider the potential for CPM when interpreting prenatal genetic screening results, especially in pregnancies complicated by placental insufficiency, which may exacerbate the apoptotic activity in placental regions, or other structural abnormalities that could alter the distribution or representation of cffDNA in maternal plasma.

#### 2.2.3. Overcoming CPM Challenges with NGS and dPCR

NGS and dPCR employ distinct strategies to address the challenges posed by CPM in NIPT. NGS leverages its genome-wide sequencing capability to detect mosaic patterns through coverage depth and genomic inconsistencies, aided by advanced bioinformatics tools. However, it is prone to both false positives, when abnormal placental regions dominate the cffDNA pool, and false negatives, when mosaic signals are too diluted for detection [51]. dPCR, by contrast, provides clearer results in cases of known abnormalities through its partitioning capability, enabling the precise detection of low-frequency mosaic signals in CPM. By isolating and amplifying specific fetal DNA fragments, it reduces maternal cfDNA interference and enhances accuracy in detecting mosaic patterns [21].

Currently, neither NGS nor dPCR can completely overcome the challenges posed by CPM, as both remain susceptible to false-positive or false-negative results. Combining the two technologies—using NGS for initial screening and dPCR for validation—offers a promising approach to mitigate CPM-related issues. Additionally, advancements in bioinformatics, artificial intelligence, and integrative methodologies may further improve the accuracy and reliability of NIPT in pregnancies complicated by CPM.

### 2.3. Maternal Influences and Gestational Dynamics in cffDNA Release

Maternal factors such as body weight, age, and lifestyle choices can significantly affect the accuracy of NIPT by influencing the FF in maternal blood. For instance, higher body weight, often measured by body mass index (BMI), correlates with an increased concentration of maternal cfDNA, which dilutes the fetal DNA fraction and raises the risk of NIPT failure due to insufficient fetal DNA. Similarly, advanced maternal age can impair placental efficiency, leading to reduced and unstable cffDNA release, further decreasing the accuracy of NIPT. In addition, unhealthy lifestyle choices like smoking exacerbate placental stress and apoptosis, increasing the release of cffDNA. However, this elevated cffDNA level may originate from damaged or stressed placental regions, introducing fragmented or low-quality DNA into the maternal circulation. Such DNA fragments often do not accurately represent the fetal genome, increasing background noise and complicating the discrimination between fetal and maternal cfDNA. This noise reduces the reliability of genetic analysis, particularly in low-FF scenarios [52,53,54]. Together, these factors contribute to variability in NIPT performance, highlighting the importance of considering maternal characteristics in the testing process.

Metabolic conditions, such as gestational diabetes and pregnancy-induced hypertension (PIH), contribute to increased placental apoptosis and ischemia, thereby elevating cffDNA levels [55]. In managing these conditions, medications such as low-molecular-weight heparin (LMWH) and aspirin are often prescribed, particularly when there is a risk of clotting or preeclampsia [56]. However, these medications can further influence cffDNA analysis by increasing the presence of smaller, guanine–cytosine-rich DNA fragments in maternal plasma, which may skew sequencing outcomes. For instance, LMWH reduces placental apoptosis, leading to lower cffDNA release and a decreased FF, potentially increasing the likelihood of NIPT failure [57,58]. Thus, though these medications help manage maternal health conditions, they can also complicate the accuracy of NIPT.

Maternal malignancies add complexity to cffDNA analysis, as tumor-derived DNA (ctDNA) can mimic fetal DNA, leading to potential false-positive or false-negative results [59]. Similarly, fetal chromosomal abnormalities, such as trisomy 21, trisomy 18, and trisomy 13, also significantly impact the FF. Trisomy 21 typically results in a higher FF, improving detection accuracy, while trisomy 18 and trisomy 13 are associated with lower FF, likely due to abnormal placental development, complicating detection and increasing the risk of false negatives [54]. In cases of multiple pregnancies, cffDNA from each fetus enters maternal circulation, increasing total cffDNA levels and complicating the identification of genetic abnormalities due to difficulties in determining the origin of the genetic material. Dilution effects, CPM, and disparities in fetal DNA contributions further elevate the risk of false-negative or false-positive results, adding complexity to prenatal diagnostics [60].

As gestational age progresses, cffDNA concentrations exhibit a positive correlation with pregnancy duration. The maturation of the placenta leads to the increased apoptosis of trophoblast cells, which release more fetal DNA into maternal circulation. Studies have shown that during early pregnancy (10 to 21 weeks), the rise in cffDNA is gradual, at approximately 0.1% per week, which can result in insufficient cffDNA levels for early detection. In contrast, during late pregnancy (after 21 weeks), the release of fetal DNA accelerates, with concentrations increasing by about 1% per week, enhancing the reliability of cffDNA-based testing in the second and third trimesters [61]. However, individual variability in cffDNA levels during early pregnancy suggests that the optimal timing for NIPT should be personalized. Although most research supports a general increase in cffDNA levels as gestation progresses, insufficient fetal DNA fractions may still occur in some women during early pregnancy, underscoring the need for individualized testing strategies.

#### NGS and dPCR: Addressing Maternal Interference Factors

Both NGS and dPCR face challenges when dealing with maternal interference factors such as high BMI, smoking, and medications like LMWH. For NGS, the primary issue is the dilution of fetal DNA signals in low-FF scenarios or high maternal cfDNA levels, which can lead to false negatives, ambiguous results, or even test failure in some cases [62]. Although advanced bioinformatics tools partially mitigate these issues by analyzing fragment size and methylation patterns, they cannot completely overcome the limitations posed by significant maternal interference. dPCR, as mentioned earlier, has advantages over NGS in handling low-FF conditions due to its targeted approach and precise quantification of specific fetal DNA fragments. However, it remains limited in detecting unexpected genetic variations and addressing complex scenarios such as CPM or twin pregnancies. Although both methods have their strengths, neither fully resolves the challenges of maternal interference, highlighting the need for further technological advancements.

## 3. From Maternal Serum Screening to NIPT with NGS and dPCR

### 3.1. Traditional Maternal Serum Screening

Fetal chromosomal aneuploidies, such as trisomy 21, trisomy 18, and trisomy 13, are common genetic disorders that can lead to intellectual disabilities and severe congenital defects in newborns [63]. The early detection of these aneuploidies is crucial for guiding prenatal care and preparing for necessary medical interventions.

Traditionally, maternal serum biochemistry screening methods, such as combined first-trimester screening (FTS) and the second-trimester quadruple test, have been widely used to assess the risk of chromosomal conditions. FTS, performed between 11 and 13 + 6 weeks of gestation, evaluates factors like maternal age, nuchal translucency (NT), pregnancy-associated plasma protein-A (PAPP-A), and free beta human chorionic gonadotropin (free β-hCG), achieving a sensitivity of 83–90% with a 5% false-positive rate for trisomy 21 [64]. The second-trimester quadruple test (15–18 weeks) includes measurements of alpha-fetoprotein (AFP), free β-hCG, unconjugated estriol (uE3), and inhibin A, with a sensitivity of 81–83% and a 5% false-positive rate for trisomy 21 [65]. Sensitivities for trisomy 18 and trisomy 13 are lower, ranging from 60 to 80% and 50 to 75%, respectively, with higher false-positive rates [66,67]. These limitations, coupled with the reliance on invasive confirmatory procedures like amniocentesis, have underscored the need for more accurate and less invasive screening methods.

### 3.2. NIPT with NGS

The advent of NIPT has revolutionized prenatal screening by directly analyzing cffDNA in maternal plasma, offering higher sensitivity and specificity. Among NIPT technologies, NGS is the most widely adopted approach, enabling the high-throughput sequencing of cffDNA. NGS begins with the extraction of cfDNA from maternal plasma, followed by library preparation and massively parallel sequencing (MPS). Millions of DNA fragments are sequenced simultaneously, generating vast data for bioinformatics analysis. Statistical models, such as z-score calculations, are applied to detect chromosomal aneuploidies with high precision [68]. NGS-based NIPT achieves remarkable accuracy, as demonstrated in a large-scale study in China involving 282,911 pregnancies, which reported a sensitivity and specificity of 99.25% and 99.98% for trisomy 21 and 98.33% and 99.98% for trisomy 18. However, for trisomy 13, the positive predictive value (PPV) is significantly lower (18.18%) due to its rarity, despite similar sensitivity and specificity [69]. With the accumulation of clinical experience and ongoing advancements in algorithms, expanded NGS-based NIPT (NIPT-plus) has been developed, enhancing its detection capabilities to include sex chromosomal aneuploidies (SCAs), rare autosomal trisomies (RATs), and copy number variations (CNVs), in addition to the common aneuploidies such as trisomy 21, trisomy 18, and trisomy 13 [70,71]. However, the low incidence of these syndromes limits their PPV, posing challenges for clinical application. To date, NGS remains the most comprehensive and reliable technique for NIPT, setting a high standard for emerging alternatives such as dPCR.

### 3.3. NIPT with dPCR

Compared to NGS, dPCR offers simpler workflows, faster data analysis, and shorter turnaround times. Its growing accuracy and the ability to operate without centralized laboratory facilities make it a promising tool for clinical departments. dPCR is particularly well suited for NIPT after the 10th week of gestation. The latest systematic review and meta-analysis [72] reported that dPCR achieved a sensitivity of 98% and specificity of 99% for detecting trisomy 21, demonstrating excellent discriminative ability (positive LR: 84.60; negative LR: 0.05). For trisomy 18, the sensitivity was 90%, and the specificity was 99.6%, indicating some challenges associated with lower FF. In the case of trisomy 13, although data were limited, dPCR showed a sensitivity of 100% and specificity of 98.2%. Despite its high accuracy in detecting specific chromosomal abnormalities, dPCR’s current clinical use remains focused on targeted testing, with broader genetic screening applications still being explored.

### 3.4. Cost-Effectiveness Comparison of NGS and dPCR

The cost of NGS-based NIPT is influenced by sequencing depth and laboratory infrastructure, typically averaging USD 570 per test, with a range of USD 200 to 1100 per test [73,74,75,76]. Key cost contributors include sequencing reagents, high-throughput sequencing platforms, bioinformatics processing, and skilled personnel.

dPCR-based NIPT remains in the research phase, with costs staying high due to the small sample size. Current estimates suggest a per-sample cost of between EUR 30 and 40, depending on the number of PCR reactions required to obtain 5900 positive droplets [77]. Running eight samples on the RainDance platform costs approximately USD 600 per run, with potential cost reductions on other dPCR platforms [78].

With large-scale implementation, the cost per dPCR test could drop below USD 100, potentially reaching the price of traditional serum biochemical tests (SBTs), which currently cost around USD 53 per test [73,79].

### 3.5. Limitations in Detecting Certain Genetic Variations

NGS is a powerful tool for broad genetic screening but has inherent limitations in detecting certain genetic variations. Standard short-read sequencing has limited sensitivity in detecting balanced translocations and inversions, as these structural variants do not involve copy number changes and are challenging to identify. While paired-end mapping and split-read analysis can provide some insights, their effectiveness is limited in complex genomic regions. Utilizing advanced structural variant detection algorithms and long-read sequencing technologies may further enhance detection accuracy [80,81]. Low-coverage sequencing approaches in NGS often struggle to detect small CNVs (<1 Mb), as these may fall below the resolution threshold of standard analytical pipelines. Increasing sequencing depth and utilizing specialized bioinformatics tools can enhance sensitivity, though these refinements are not yet standard in clinical NIPT [82]. Another limitation is the challenge in phasing alleles directly using short-read NGS, which is crucial for identifying compound heterozygous mutations in recessive diseases. Trio-based sequencing and haplotype inference methods can help address this limitation, while long-read sequencing offers direct phasing capabilities but remains costly and is not yet widely used in routine NIPT practice [83]. Complex structural variations, such as segmental duplications and rearrangements with undefined breakpoints, also pose challenges. Misalignment of sequencing reads in repeat-rich regions can lead to false variant calls, further complicating accurate variant interpretation [84,85,86,87].

Similarly, dPCR is constrained in its ability to detect genetic variations beyond predefined loci. It is unsuitable for genome-wide screening or identifying novel mutations and is primarily used for detecting large CNVs at predefined loci, particularly those associated with known microdeletion and microduplication syndromes [88]. However, it cannot reliably assess CNVs with undefined breakpoints or unbalanced translocations [89,90]. Additionally, dPCR does not provide phased allele information, which is necessary for distinguishing compound heterozygous variants in recessive conditions. Allele dropout (ADO) increases the risk of false negatives in heterozygous mutation detection, particularly when DNA input is low or degraded. This phenomenon arises from biased amplification during PCR, but optimizing DNA purification, increasing template input, and refining assay design can help mitigate its impact [91,92].

## 4. The Working Principle of dPCR Technology for NIPT

dPCR uses endpoint detection to calculate the copy number of target sequences, enabling precise absolute quantification without the need for reference genes or standard curves. Unlike traditional PCR, it bypasses the cycle threshold (Ct values), thereby minimizing the impact of amplification efficiency on the final results [21]. Its high tolerance to PCR inhibitors, achieved through reaction partitioning and optimized systems, further enhances accuracy and reproducibility, even in challenging sample matrices. Based on the form of the reaction unit, dPCR systems can be divided into three main categories, microfluidic, droplet-based and chip-based dPCR systems, each with distinct benefits suited to specific applications [93].

### 4.1. Microfluidic Digital PCR (mdPCR)

mdPCR leverages microfluidic channel partitioning to separate DNA templates into nanoliter or smaller reaction units, enabling precise absolute quantification. Although microfluidic technology allows for precise control, managing and processing droplets, especially integrating them with the PCR reaction system, remains challenging. Alternative methods with simpler workflows and improved scalability are gaining traction, gradually reducing the reliance on mdPCR in some applications [94]. Moreover, the commercial prominence of mdPCR has declined over time, with systems like RainDrop being integrated into broader platforms following the acquisition of RainDance Technologies by Bio-Rad. As a result, mdPCR is now primarily referenced as a foundational technology rather than a widely used platform.

### 4.2. Droplet-Based Digital PCR (ddPCR)

ddPCR utilizes an oil-in-water technique to partition samples into thousands of nanoliter-sized droplets. Each droplet may contain zero, one, or multiple target nucleic acid molecules [95].

**Figure 2:**
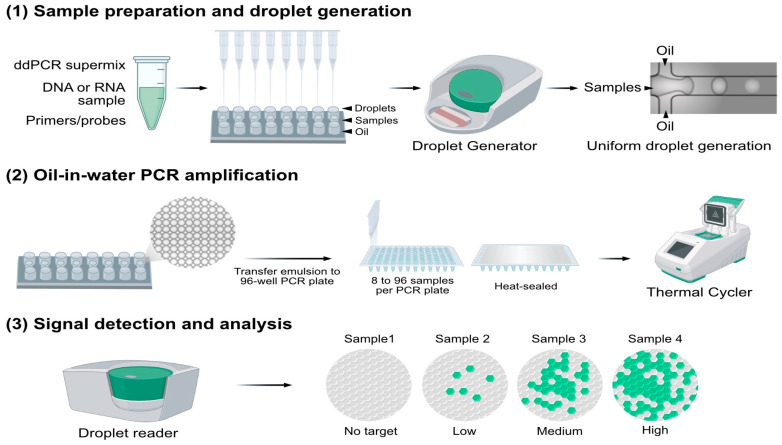
Overview of Bio-Rad’s QX200 digital droplet PCR (ddPCR) system (Bio-Rad Laboratories, Inc., Hercules, CA, USA) workflow. Redrawn based on content from Droplet DigitalTM PCR Applications Guide and QX200TM Droplet Generator Instruction Manual.

Taking Bio-Rad’s QX200 ddPCR system (Bio-Rad Laboratories, Inc., Hercules, CA, USA) as an example [96], the main steps are as follows: (1) Sample preparation and droplet generation: Samples and oil are loaded into an 8 × 3 droplet generation cartridge, with one row containing the sample and another containing the oil. A droplet generator produces approximately 20,000 droplets per sample. (2) Oil-in-water PCR amplification: The generated droplets are transferred to a microplate. The microplate is then placed into a PCR amplification machine, where each droplet undergoes 40 cycles of thermal cycling for PCR amplification. (3) Signal detection and analysis: The droplets are transferred to a droplet reader, which employs flow cytometry to detect fluorescence signals after PCR amplification. The results are then quantified using the Poisson distribution principle. The procedure is represented in Figure 2.

### 4.3. Chip-Based Digital PCR (cdPCR)

cdPCR utilizes integrated fluidic circuit technology, where numerous microchannels and microchambers are etched onto silicon wafers or quartz glass. By controlling various valves, the flow of solutions is precisely regulated within these microstructures, dividing the sample liquid into uniform nanoliter-sized reaction wells for digital PCR. This process enables the absolute quantification of target molecules [97].

**Figure 3:**
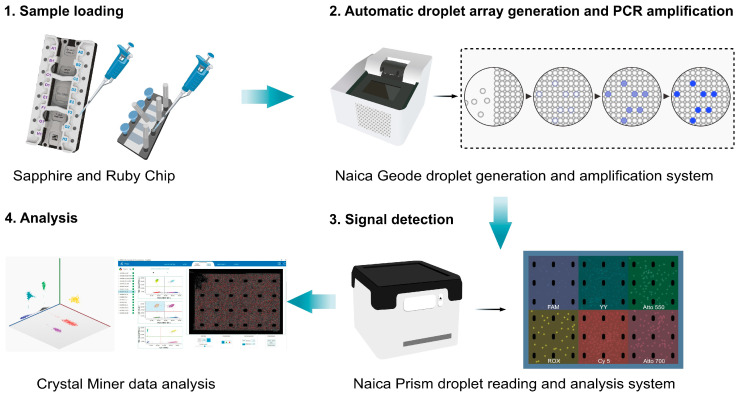
Overview of the Stilla Technologies’ Naica Crystal Digital PCR system (Stilla Technologies, Villejuif, France) workflow. Adapted from the Stilla Naica System Brochure, with permission from Stilla Technologies.

Taking Stilla Technologies’ Naica Crystal Digital PCR system (Stilla Technologies, Villejuif, France) as an example [98], the main steps are as follows: (1) Sample preparation: the sample and PCR reaction mixture are added to the microfluidic chip. (2) Droplet generation and PCR amplification: The Naica Geode system generates an array of 20,000 to 30,000 uniform droplets within microfluidic channels, each serving as an individual reaction compartment. The system then performs automated PCR amplification on these droplets, with precise control over temperature cycling to maintain stable and consistent droplet formation. Droplet generation and thermal cycling are integrated into a single, streamlined workflow. (3) Signal detection: the chip is placed in the Prism system, which uses six-channel fluorescence detection to count positive and negative droplets. (4) Analysis: the Poisson distribution is used to calculate the absolute copy number of the target gene. The procedure is represented in Figure 3.

cdPCR supports high-level multiplex PCR technology, allowing the detection of up to eight copy number variations (CNVs), five mutations, or four gene expressions in a single reaction.

## 5. The Role of dPCR in Prenatal Testing

The application of dPCR in prenatal testing can be categorized as either NIPT/NIPS or Non-Invasive Prenatal Diagnosis (NIPD), depending on the target being analyzed. When dPCR is used to detect chromosomal aneuploidies or microdeletions/microduplications, it functions primarily as a screening tool to assess whether the fetus may have an abnormal number of chromosomes. This testing is categorized as screening rather than diagnostic because it analyzes a mixture of maternal and fetal cfDNA, which can result in false-positive or false-negative outcomes. Biological factors such as the proportion of fetal to maternal cfDNA, or placental mosaicism, may affect the accuracy of chromosomal abnormality detection. As a result, even if dPCR indicates a risk of aneuploidy or microdeletion/microduplication, confirmatory tests like amniocentesis or CVS are required for diagnosis. Therefore, in these cases, dPCR serves as a screening tool, known as NIPT/NIPS.

When dPCR is used to detect monogenic diseases, it analyzes specific gene mutations in fetal cfDNA to distinguish fetal from maternal genetic material. For paternal mutations or de novo variants, detection is relatively straightforward. In contrast, detecting maternally inherited mutations often relies on the quantitative analysis of allelic ratios using methods like the relative mutation dosage (RMD). Combined with the ability to identify fetal-specific variants, such as paternal alleles and de novo mutations, dPCR enables the precise evaluation of fetal genetic material. Unlike chromosomal aneuploidy detection, which heavily relies on fetal cfDNA proportion, monogenic disease detection focuses on identifying specific genetic changes, reducing the need for invasive confirmation in most cases. This precision makes dPCR a valuable tool for NIPD, offering reliable insights into fetal genetic conditions.

## 6. Applications of dPCR in NIPT for Chromosomal Aneuploidy

### 6.1. Early Applications of dPCR in NIPT (2007–2015)

Between 2007 and 2015, dPCR made significant strides in detecting chromosomal aneuploidies. Lo YM et al. (2007) demonstrated dPCR’s ability to precisely quantify chromosomal imbalances by using digital RNA SNP analysis combined with the relative chromosomal dosage (RCD) to detect trisomy 21 through the analysis of SNPs in the *PLAC4* gene [99]. Fan HC et al. (2007) further validated dPCR’s potential for large-scale testing using a high-throughput microfluidic chip capable of 9180 parallel nanoliter-scale reactions. They applied this method to mixed DNA samples with varying proportions of trisomy 21 (30–60%) and normal DNA, achieving 95% confidence in distinguishing trisomy 21 samples with as low as 40% trisomy DNA from normal samples [16]. Lun FM et al. (2008) demonstrated that mdPCR could accurately quantify fetal DNA in maternal plasma when its fractional concentration was as low as 5% [100]. These early studies established dPCR as a reliable tool for trisomy 21 detection, laying the foundation for broader NIPT applications (Table 1).

### 6.2. Advances in dPCR and the Development of Multiplex Detection (2015–2019)

From 2015 to 2019, major improvements were achieved in dPCR technology, particularly in multiplex detection. Researchers began detecting multiple chromosomal loci simultaneously, improving efficiency and cost effectiveness. El Khattabi LA et al. (2016) used multiplex dPCR to detect chromosome 21 copy numbers, achieving 94% sensitivity and 98% specificity [77]. This mirrored earlier findings by Lun FM et al. (2008) but marked a significant improvement in multiplex capabilities. Xu S et al. (2016) detected trisomy 21 by quantifying segmental duplication (SD) markers, detecting fetal DNA concentrations as low as 10% [101]. Additionally, Li W et al. (2018) analyzed the ratios of the *HLCS* gene and rs6636 SNP, correctly identifying all euploid cases while misclassifying only two trisomy 21 cases [102].

Lee SY et al. (2015) detected trisomy 21 and 18 using just 1 to 2 mL of maternal plasma, with accuracy rates comparable to NGS [103]. By 2018, Lee SY et al. had improved their method by incorporating a size-selection technique to enrich smaller fetal DNA fragments, achieving 100% sensitivity for trisomy 21 and 99.64% specificity for trisomy-negative samples [104]. These advancements significantly improved the overall accuracy and sensitivity of dPCR, positioning it as a strong contender for clinical application in NIPT (Table 1).

### 6.3. Clinical Validation of dPCR in NIPT (2019–2021)

Between 2019 and 2021, dPCR underwent extensive clinical validation. Tan C et al. (2019) used multiplex dPCR to test 60 clinical plasma samples for trisomy 21 and 18, achieving 100% concordance with NGS results [105]. Haidong W et al. (2020) developed the iSAFE NIPT method, which applied dPCR to 269 plasma samples, detecting trisomy 13, 18, and 21 with 100% sensitivity and specificity for trisomy 21 [106]. Chen X et al. (2021) further optimized probe design by combining segmental duplication markers with computational techniques, enhancing accuracy even in low-fetal-DNA samples [107]. These studies showed that dPCR is comparable to NGS in sensitivity and specificity, but with faster processing time and lower costs, making it ideal for resource-limited clinical environments (Table 1).

### 6.4. Technological Innovations and Future Directions of dPCR (2022–Present)

Since 2022, dPCR technology has focused on further innovations, including enhanced multiplex detection, improved sample handling, and cost efficiency. These advancements aim to address existing limitations and broaden dPCR’s applications in chromosomal screening. Ramesh M et al. (2023) employed the QX600 multiplex ddPCR system to detect aneuploidies across multiple chromosomes, achieving high concordance with NGS and detecting aneuploidies at fetal fractions as low as 4% [108]. Dai P et al. (2022) demonstrated that ddPCR achieved high sensitivity for detecting trisomy 21, 18, and 13. However, false positives, particularly for trisomy 21 and 13, affected the overall specificity. Despite this, the test showed strong performance, but further refinement is required to minimize false positives and improve accuracy [73]. Lassakova S et al. (2023) validated ddPCR with locked nucleic acid (LNA) probes for low-fetal-DNA samples, achieving 100% sensitivity and specificity for trisomy 21 [109] (Table 1).

Although dPCR offers advantages in cost and speed, further development is needed to extend its application to comprehensive chromosomal aneuploidy screening. Priorities include standardizing detection methods, expanding multiplex capabilities, improving sample handling, and integrating bioinformatics for better probe design. These advancements could establish dPCR as a reliable complement to NGS in clinical chromosomal screening, especially in underfunded regions.

Table 1 summarizes the key characteristics and findings of dPCR studies on NIPT for chromosomal aneuploidies.

## 7. Clinical Applications of dPCR in NIPT for Chromosomal Microdeletions and Microduplications

Chromosomal microdeletions and microduplications involve the loss or duplication of small chromosomal segments, typically ranging from 1 kilobase pair (kb) to 5 megabase pairs (Mb). These subtle genetic alterations, known as copy number variations (CNVs), can affect one or more genes, leading to a broad spectrum of clinical disorders with phenotypes ranging from mild to severe [110]. CNVs are implicated in various syndromes, including 22q11.2 deletion syndrome (also known as DiGeorge syndrome, velocardiofacial syndrome, and CATCH-22) [111], as well as Prader–Willi, Angelman, and 1q21.1 microduplication syndromes, etc. [112,113]. These examples illustrate the broad clinical spectrum of CNVs, emphasizing the need for accurate detection methods for effective diagnosis and management.

Among these conditions, 22q11.2 deletion syndrome has received the most research attention, partly due to its higher prevalence—approximately 1 in 4000 live births—making it the most common microdeletion syndrome [111]. The broad and well-characterized phenotypic spectrum of 22q11.2 deletion syndrome has established it as a reference point for CNV research. In contrast, rarer syndromes such as 1q21.1 microduplication and Prader–Willi syndrome exhibit more variable or subtle clinical manifestations, complicating diagnosis and limiting the number of systematic studies, particularly in the context of NIPT.

Although detecting microdeletions and microduplications is clinically important, the use of dPCR in NIPT for this purpose remains limited. One key challenge is detecting these small genetic changes in the low concentrations of cffDNA in maternal plasma. Additionally, the variability in clinical outcomes complicates prenatal decision-making, as not all cases result in severe conditions. This variability often leads to diverse decisions about pregnancy continuation, shifting the research focus to postnatal populations, where the disorders are more readily observable in newborns and children.

The survival of many individuals with disorders like 22q11.2 deletion syndrome into childhood provides researchers with larger cohorts to study, enabling the validation of detection methodologies [114]. While dPCR has shown exceptional performance in the postnatal detection of aneuploidies and CNVs, its application in NIPT for detecting chromosomal microdeletions and microduplications is still in the early stages of development.

In the study by Wang J et al., a dPCR-based NIPT was developed to detect fetal 22q11.2 microdeletion/duplication syndrome. Six detection sites were targeted with corresponding probes, and a blind test was conducted on 130 maternal plasma samples, 15 of which were confirmed to carry fetal 22q11.2 microdeletion/duplication [115]. The dPCR method successfully detected eleven out of the fifteen affected samples, while four cases were missed. Among the 115 normal samples, 111 were accurately identified as unaffected. The assay demonstrated a sensitivity of 73.3% and a specificity of 96.5%, with positive and negative predictive values of 73.3% and 96.5%, respectively. These results suggest that although the assay shows potential in detecting 22q11.2 microdeletion/duplication, further optimization is required to improve its sensitivity.

Several issues were identified in the study that contributed to detection failures, including low concentrations of cffDNA, which reduced detection accuracy, and confined placental mosaicism (CPM), where differences between placental and fetal DNA led to both false positives (FPs) and false negatives (FNs). Technical limitations, such as suboptimal probe design, target site selection, and variations in sample quality and cffDNA microheterogeneity, also played a role. To improve detection rates, advancements like enhanced cffDNA extraction techniques, improved probe designs, regular calibration of dPCR instruments, and comprehensive studies to address CPM are recommended to increase sensitivity and reliability (Table 2).

Table 2 summarizes the key characteristics and findings of dPCR studies on NIPT for chromosomal microdeletions and microduplications.

## 8. Practical Applications of dPCR in NIPT for Monogenetic Disease

Monogenic diseases, caused by mutations in a single gene, affect approximately 2% of individuals and can be inherited in various ways, including autosomal dominant, autosomal recessive, and X-linked patterns [116]. dPCR provides precise allele discrimination, enabling accurate differentiation between maternal and paternal mutations, and is particularly effective in detecting rare and low-frequency mutations. This makes it a promising tool for NIPT. The following sections will focus on dPCR’s clinical applications in NIPT across different fetal systems, illustrating its potential in prenatal diagnosis.

### 8.1. Fetal Blood System Monogenetic Diseases

Fetal blood system monogenetic diseases are caused by genetic mutations or hereditary factors and can result in conditions such as severe anemia, jaundice, heart problems, and impaired growth. For example, severe cases of thalassemia can lead to heart failure, while untreated hemophilia can cause life-threatening bleeding. Early diagnosis is critical for managing these conditions to prevent complications and improve the quality of life for affected individuals [117,118,119].

#### 8.1.1. Thalassemia

Thalassemia is a hereditary blood disorder caused by mutations in the α-globin or β-globin chains, resulting in impaired hemoglobin production and severe anemia. Inherited in an autosomal recessive manner, the disease is particularly prevalent in regions such as Southeast Asia and the Mediterranean, where carrier rates can reach up to 30% [120,121,122].

Research studies have steadily advanced the application of dPCR in NIPT for thalassemia. Charoenkwan P et al. conducted an early study employing nanoplate-based dPCR to diagnose β-thalassemia in 35 at-risk couples between 12 and 18 weeks of pregnancy [123]. The technique achieved a sensitivity of 100% and specificity of 92.3% in detecting paternal mutant alleles in affected fetuses. Notably, the ratio of mutant to wild-type β-globin alleles in maternal plasma served as a reliable indicator for diagnosing fetal inheritance of maternal mutations. Subsequent research by Sawakwongpra K et al. expanded the use of ddPCR to detect both α- and β-thalassemia [124]. Although the study demonstrated high accuracy for the -SEA deletion, it faced challenges in detecting variants such as hemoglobin E (HbE) and the codon 41/42 (-CTTT) mutation, indicating that further assay refinements are needed for broader application.

Building on these advancements, Suwannakhon N et al. applied ddPCR to detect β-thalassemia mutations in 42 at-risk couples [125]. In addition to identifying paternal mutations in fetal DNA, the study provided insights into maternal inheritance patterns, with 10 of 22 paternally inherited β-thalassemia-positive cases identified as compound heterozygotes or homozygotes for β-thalassemia. D’Aversa E et al. conducted a study using 52 maternal plasma samples (5th to 39th week) to detect β+IVSI-110 and β039 mutations. The analysis successfully identified paternally inherited mutations in 23 samples and classified maternally inherited mutations in 30 heterozygous mothers (N/M) based on M/N allelic ratios. The method accurately classified 51 of 52 samples, showing suitability for the prenatal detection of β-thalassemia mutations as early as the 7th gestational week [126].

These studies highlight the advancements in dPCR technology for non-invasive thalassemia screening, offering increased accuracy and potential for routine clinical use, reducing the need for invasive procedures, and paving the way for broader clinical adoption (Table 3).

#### 8.1.2. Sickle Cell Anemia

Sickle cell anemia is a hereditary blood disorder caused by a mutation in the β-globin gene, resulting in the production of abnormal hemoglobin S (HbS) [135]. This leads to rigid, sickle-shaped red blood cells, causing vessel blockages, pain, and increased infection risk. The condition is inherited in an autosomal recessive manner and is prevalent among populations of African, Mediterranean, Middle Eastern, and Indian descent [136].

Barrett AN et al. utilized cdPCR with Minor Groove Binder (MGB) TaqMan probes to distinguish between normal hemoglobin A and mutated HbS alleles [127]. For male fetuses, the Y chromosome-specific marker DYS14 was used, while a biallelic indel marker was employed for female fetuses. The study accurately identified the sickle cell genotype in 82% of male and 75% of female fetuses when fetal DNA concentrations were at least 7%. Although promising, the method requires further refinement in fetal DNA measurement and marker development, particularly for female fetuses (Table 3).

#### 8.1.3. Hemophilia

Hemophilia is a hereditary bleeding disorder caused by mutations in the genes responsible for producing clotting factors, primarily factor VIII (hemophilia A) or factor IX (hemophilia B). These mutations lead to prolonged bleeding and spontaneous bleeding episodes, particularly in joints and muscles. Hemophilia is inherited in an X-linked recessive manner, predominantly affecting males, while females are typically carriers [137]. Early diagnosis and treatment with clotting factor concentrates are crucial for managing bleeding episodes, preventing joint damage, and improving the quality of life for individuals with hemophilia [138].

Tsui NB et al. used microfluidic dPCR and the relative mutation dosage (RMD) method to determine whether fetuses had inherited hemophilia mutations by measuring the overexpression of either the mutant or wild-type allele [128]. In plasma samples from twelve pregnant women, including seven hemophilia carriers with male fetuses (three carriers of hemophilia A, four carriers of hemophilia B), the test accurately identified fetal hemophilia genotypes as early as the 11th week of gestation. Hudecova I et al. further advanced this approach by combining ddPCR with targeted massive parallel sequencing (MPS) to assess (Factor VIII) *F8* and (Factor IX) *F9* gene mutations in 15 fetuses from hemophilia carriers [129]. Their study successfully identified known sequence variants and, using MPS with haplotype analysis, non-invasively detected complex intron 22 homolog-related (int22h-related) inversions in the *F8* gene, broadening the diagnostic capabilities for hemophilia (Table 3).

### 8.2. Fetal Skeletal Muscle System Monogenetic Diseases

Fetal skeletal muscle system genetic diseases, such as Duchenne muscular dystrophy (DMD), spinal muscular atrophy (SMA), achondroplasia, and osteogenesis imperfecta, are inherited disorders that affect muscle and bone development, leading to conditions like muscle weakness and skeletal deformities. While NIPT technologies are increasingly being applied for detecting these conditions, progress is still ongoing. Notably, dPCR has shown potential in NIPT for diseases like achondroplasia and SMA, offering early, accurate detection through cffDNA analysis in maternal plasma [139].

#### 8.2.1. Achondroplasia

Achondroplasia is the most common form of dwarfism, characterized by short stature, disproportionately short limbs, and a larger forehead. It is caused by a mutation in the *FGFR3* gene, which affects bone and cartilage growth. Inherited in an autosomal dominant manner, only one copy of the mutated gene is needed for the condition to occur [140]. With proper medical care and support, individuals with achondroplasia can lead healthy, productive lives [141].

Orhant L et al. employed ddPCR combined with mini-sequencing to target specific mutations in the *FGFR3* gene, which are known to cause achondroplasia [130]. The study included 26 maternal plasma samples from women identified as high-risk due to third-trimester ultrasound findings. The method successfully identified five fetuses affected by achondroplasia. Pacault M et al. tested fifty-nine cases with ultrasound findings suggestive of skeletal dysplasia, resulting in the detection of nineteen cases with the *FGFR3* c.1138G>A mutation and one case with the rarer c.1138G>C mutation, both associated with achondroplasia [131]. No *FGFR3* mutations c.742C>T, c.1118A>G, or c.1948A>G, which are linked to thanatophoric dysplasia (TD), were detected. The dPCR results were fully consistent with those from invasive prenatal testing, with no inconclusive outcomes (Table 3).

#### 8.2.2. Spinal Muscular Atrophy (SMA)

Spinal muscular atrophy (SMA) is a genetic disorder characterized by progressive muscle weakness and wasting, resulting from the loss of motor neurons in the spinal cord. It is mainly caused by mutations in the *SMN1* gene, which lead to insufficient production of the survival motor neuron (SMN) protein essential for motor neuron function. SMA is inherited in an autosomal recessive manner, meaning a child must inherit a copy of the mutated gene from both parents to be affected [142].

Wei X et al. accurately detected fetal *SMN1* copy number by targeting the sixth nucleotide in exon 7, the most common deletion mutation associated with SMA, achieving 100% concordance with traditional multiplex ligation-dependent probe amplification (MLPA) testing [78] (Table 3). Building on this, Tan C et al. developed a single-tube multiplex dPCR assay that simultaneously detects *SMN1* and *SMN2* copy numbers, targeting deletions in exons 7 and 8, which are critical for determining SMA severity [143]. Tested on various clinical samples, including peripheral blood, amniotic fluid, and buccal swabs, the assay demonstrated high accuracy and reliability across all sample types. Although this assay was not directly applied in NIPT, it shows significant potential for future NIPD applications, given its ability to accurately detect genetic variations in different sample types relevant to prenatal diagnostics.

Notably, the ability to predict disease severity based on the *SMN2* copy number adds valuable prognostic insight. Both studies underscore the potential of dPCR as a highly accurate, non-invasive diagnostic tool for SMA, capable of detecting both *SMN1* deletions and *SMN2* copy number variations. The technology offers a safer and faster alternative to invasive methods like amniocentesis, making it highly suitable for broader clinical use in SMA diagnosis and carrier screening.

### 8.3. Fetal Auditory System Monogenetic Disorders

#### Genetic Deafness

Fetal auditory system monogenetic disorders primarily involve inherited hearing loss caused by single-gene mutations, with genetic deafness being the most prevalent example. Mutations in genes like *GJB2*, *MYO7A*, and *OTOF* commonly lead to partial or profound hearing loss, which can affect one or both ears. These disorders follow different inheritance patterns, such as autosomal recessive, autosomal dominant, and X-linked. The early detection of genetic deafness is crucial, as timely interventions, including the use of cochlear implants or hearing aids, can significantly enhance the quality of life for affected individuals [144].

Chang MY et al. developed a method for detecting autosomal recessive homozygous point mutations, specifically targeting mutations linked to congenital hearing loss, such as *GJB2* and *SLC26A4* [132]. Their one-step protocol, which removes the need to estimate fetal DNA fraction, integrates chi-squared analysis with Bayesian statistical methods for accurate fetal genotype prediction. The approach was successfully applied to three families carrying hearing-loss-related mutations (*GJB2* c.235delC and *SLC26A4* IVS7-2A>G), demonstrating its effectiveness in NIPD (Table 3).

### 8.4. Fetal Respiratory and Digestive System Monogenetic Diseases

Fetal respiratory and digestive system monogenetic diseases include conditions such as cystic fibrosis (CF), alpha-1 antitrypsin deficiency, and congenital diaphragmatic hernia [145,146,147]. Among them, CF is the most extensively studied using dPCR due to its well-characterized *CFTR* gene mutations, allowing for precise targeted detection. In contrast, other conditions are either rarer or involve more complex genetic mechanisms, which limits the current application of dPCR in their diagnosis.

#### Cystic Fibrosis

Cystic fibrosis (CF) is a genetic disorder affecting the lungs and digestive system. It causes thick, sticky mucus buildup, leading to respiratory infections and digestive issues. CF is caused by mutations in the *CFTR* gene and is inherited in an autosomal recessive manner. Management includes medications, physiotherapy, and nutritional support to improve quality of life [148].

Gruber A et al. successfully detected paternally inherited *CFTR* mutations in fetal DNA by analyzing cffDNA from maternal plasma [133]. Using ddPCR, they differentiated fetal DNA from maternal DNA based on the unique paternal allele, accurately counting mutant and wild-type alleles in individual droplets. This method detected only the paternal *CFTR* mutation, excluding maternal DNA, and demonstrated 100% accuracy in identifying the mutation in 15 high-risk pregnancies. The results confirmed the reliability of ddPCR for NIPD compared to traditional invasive testing methods.

Pacault M et al. investigated pregnancies at risk of CF by detecting specific *CFTR* mutations as c.1521_1523del (ΔF508) [131]. Taqman probes were used to differentiate between mutant and normal alleles. The study accurately identified *CFTR* mutations, with fetal DNA fractions ranging from 1.6% to 16.4%. ddPCR showed high specificity in distinguishing between overlapping *CFTR* mutations, making it a reliable method for NIPD. Similarly, Debrand E et al. conducted a study focusing on the detection of the ΔF508 mutation in three pregnancies at high risk for CF due to compound heterozygosity [134]. Taqman probes targeting exon 11 of the *CFTR* gene were used to quantify the ratio of mutant to normal alleles. When the proportion of mutant alleles exceeded background levels (typically 0.1% or lower), it indicated that the fetus had inherited the paternal mutation. ddPCR accurately identified the ΔF508 mutation in all three cases, detecting fetal DNA fractions as low as 1% of the total DNA in maternal plasma.

Both studies utilized ddPCR with Taqman probes to detect *CFTR* mutations, particularly the ΔF508 mutation, through quantitative analysis of mutant and normal alleles in cffDNA. Although both employed similar methods, Pacault’s study involved a larger sample size and focused on distinguishing overlapping mutations, whereas Debrand’s study optimized the detection sensitivity for low fetal DNA fractions (Table 3).

### 8.5. Fetal Nervous System Monogenetic Diseases

Fetal nervous system monogenetic diseases include conditions such as neurofibromatosis type 1 (NF1), Tay–Sachs disease, and Rett syndrome [149]. These disorders disrupt neural development, leading to significant neurological impairments that may manifest prenatally or in early childhood. Currently, only NF1 has been studied using dPCR. NF1 is an autosomal dominant monogenic disorder caused by mutations in the *NF1* gene [150].

#### Neurofibromatosis Type 1 (NF1)

Research studies detected *NF1* mutations through NIPD by targeting paternally inherited mutations in maternal blood, allowing for a clear distinction between fetal and maternal DNA [131,133]. This approach is particularly crucial for autosomal dominant conditions like NF1, where the paternal allele can be traced in at-risk pregnancies.

Gruber A et al. studied four pregnancies at risk for NF1 using hydrolysis probes targeting the paternal *NF1* mutation [133]. This method accurately distinguished between paternal mutations and maternal DNA by counting mutant and wild-type alleles separately, achieving high sensitivity in detecting *NF1* mutations in fetal fractions as low as 1.5%.

Pacault M et al. conducted a three-year study using ddPCR to test 24 samples for paternal autosomal dominant single-gene disorders, including NF1 [131]. By designing probes to differentiate between mutant and wild-type alleles, they successfully detected paternal *NF1* mutations, with fetal DNA fractions ranging from 1.8% to 12.5%. Despite challenges in detecting the *NF1 c.2033dup* variant due to assay design difficulties, ddPCR proved highly feasible and accurate for *NF1* testing (Table 3).

Table 3 summarizes the key characteristics and findings of dPCR studies on NIPT and NIPD for monogenetic diseases.

## 9. Challenges in Detecting Maternally Inherited Mutations and the Role of dPCR

Detecting maternally inherited monogenic diseases through NIPT is particularly challenging due to the shared genetic information between mother and fetus. In maternal blood, cffDNA is mixed with a large amount of maternal cfDNA, and the low proportion of cffDNA complicates distinguishing fetal mutations from the maternal background. This challenge becomes even more pronounced when the mother carries the same genetic mutation, as both maternal and fetal cfDNA contribute identical copies of the mutation, making differentiation highly complex.

To address these complexities, NGS has incorporated advanced methods like the Relative Haplotype Dosage (RHDO), which improves the ability to differentiate fetal-specific alleles from the maternal background. The RHDO achieves this by constructing parental haplotype maps, performing deep sequencing of cfDNA in maternal plasma, and analyzing the relative abundance of haplotypes. By detecting shifts in haplotype representation caused by fetal contributions, the RHDO enables the precise identification of haplotypes inherited by the fetus. This method is particularly effective in distinguishing shared mutations between the mother and fetus, significantly improving detection sensitivity and accuracy despite the challenges posed by low fetal fractions and overlapping genetic material [151].

In contrast, dPCR provides a more targeted and efficient solution for detecting maternally inherited mutations in certain scenarios. Using the RMD method, it compares the abundance of wild-type and mutant alleles in maternal plasma by analyzing shifts caused by fetal cfDNA. If the fetus inherits the wild-type allele, its proportion increases due to additional fetal cfDNA. Conversely, if the fetus inherits the mutant allele, the proportion of mutant alleles rises accordingly. The RMD accurately quantifies these changes in allele proportions to determine which allele the fetus has inherited from the mother [152]. Although dPCR is highly effective for detecting single mutations, the RMD method is less suitable for screening multiple mutations simultaneously and may face difficulties with very low fetal fractions. More comprehensive methods like the RHDO offer broader detection but are more complex and expensive.

## 10. Prospects

dPCR is set to transform NIPT and NIPD by advancing early disease detection and fetal health monitoring. Future developments will aim to expand its detection range and accuracy, enabling more precise personalized prenatal care.

Integrating dPCR with NGS, artificial intelligence (AI), and machine learning will further enhance diagnostic precision. AI-driven analysis can facilitate automated data interpretation, may reduce false positives and negatives, and can optimize FF assessment, ultimately improving the clinical utility of dPCR in NIPT [153]. Moreover, AI-based predictive models could aid in stratifying high-risk pregnancies by integrating genomic, clinical, and imaging data, thereby enhancing risk assessment and personalized patient management. The integration of AI with dPCR could also contribute to the development of real-time, automated decision-support tools for obstetricians, reducing reliance on invasive procedures and improving overall prenatal diagnostic efficiency [154,155].

Beyond detecting genetic disorders, dPCR has the potential to identify fetal-specific biomarkers, such as cfDNA and microRNA, providing deeper insights into fetal responses to maternal health conditions. Building on existing research, these advancements could improve the prediction and management of maternal–fetal complications, such as placental dysfunction, fetal growth restriction, and preeclampsia, by enabling earlier detection, more precise monitoring, and targeted interventions.

Integrating dPCR with advanced microfluidic systems has the potential to streamline NIPT workflows by automating cfDNA processing and improving reaction efficiency [156]. Additionally, combining dPCR with microfluidics and AI-driven automation may enhance sample throughput, minimize handling errors, and reduce turnaround times, making high-precision NIPT more accessible and scalable. These innovations could also enable continuous fetal health monitoring, allowing real-time assessments and early intervention for conditions like intrauterine infections and immune-related disorders. Furthermore, microfluidic platforms integrated with AI-assisted analytics could improve cfDNA enrichment efficiency, allowing for lower input sample requirements and enhancing the sensitivity of dPCR for detecting rare fetal genetic abnormalities [157].

Further advancements in single-cell dPCR have enhanced precision in detecting rare fetal genetic variations and mosaicism by analyzing fetal DNA at the single-cell level [158]. Unlike conventional dPCR, which measures cfDNA in maternal plasma, single-cell dPCR reduces maternal DNA interference and improves the detection of low-abundance variants, monogenic disorders, and confined placental mosaicism [159,160]. These improvements make single-cell dPCR a promising tool for increasing the accuracy of NIPT. Looking forward, the integration of single-cell dPCR with AI-driven bioinformatics and microfluidic-based single-cell isolation could further refine NIPT accuracy, particularly for complex genetic conditions such as de novo mutations and low-level fetal mosaicism. This combined approach may also expand the clinical applicability of dPCR by enhancing sensitivity in detecting fetal abnormalities that might be missed by conventional NIPT.

Ultimately, dPCR’s future aligns with personalized medicine, offering precise genetic insights to tailor prenatal care and improve outcomes for both mothers and their babies. The convergence of dPCR with AI, microfluidics, and other emerging technologies will likely drive the next generation of NIPT, making it more efficient, cost-effective, and clinically actionable.

## 11. Conclusions

This review consolidates existing research on the applications of dPCR in NIPT, providing a comprehensive and structured summary of its current strengths and limitations. By systematically categorizing its utility across chromosomal aneuploidies, microdeletions/duplications, and monogenic diseases, it lays a robust foundation for understanding the clinical potential of dPCR. It highlights the continuous advancements in technologies aimed at addressing challenges such as the detection of low-abundance fetal DNA, the precise quantification of rare mutations, and the integration of dPCR with computational tools to enhance assay design. These developments underscore the intricacies of the field while unveiling opportunities for further innovations to improve diagnostic accuracy and broaden clinical applications.

Looking ahead, dPCR’s high sensitivity, specificity, and multiplexing capabilities position it as a transformative tool in NIPT. Its ability to handle complex samples while minimizing false positives and negatives enhances its reliability, making it an essential component of personalized prenatal care. With continued refinements in technology and expanded clinical validation, dPCR is poised to set a new benchmark in NIPT, advancing maternal–fetal healthcare and addressing the limitations of current diagnostic approaches.

## Figures and Tables

**Table 1:** Summary of dPCR studies on NIPT for detection of chromosomal aneuploidies.

Author (Year)	Focus of Study	Method Used	Sample Information	Key Findings/Implications	Diagnostic Accuracy	Major Limitations
Lo YM et al. (2007) [99]	T21	ddPCR	Normal = 9, T21 plasma = 4; normal = 2, T21 placenta = 2; GA: NA	Identified chromosomal imbalances through SNPs in the *PLAC4* gene and chromosome dosage	The RNA and DNA from both plasma and placental samples were all correctly classified	Requires high FF (≥25%); applicable only to SNP-based detection; labor-intensive process
Fan HC et al. (2007) [16]	T21	mdPCR	Human genomic DNA from normal and T21 cell lines; GA: NA	Detected T21 by amplifying and quantifying single DNA molecules in mixed samples	T21 can be distinguished with maternal contamination or fetal mosaicism	High DNA input (≥10^3^ copies required); low sensitivity (≥10% fetal DNA needed)
El Khattabi LA et al. (2016) [77]	T21	ddPCR	Normal = 192, T21 = 21; GA: 9–37 weeks	Detected T21 with 5% fetal DNA content	Sensitivity = 94%; specificity = 98%	Exclusion of 9 low-quality samples (4 T21) may overestimate sensitivity; no FF estimation (impacts reliability)
Xu S et al. (2016) [101]	T21	cdPCR	Normal = 12, T21 = 3; GA: NA	Quantified small increases in Chr21 with 10% fetal DNA content	Accuracy = 100%	Inferred FF: reducing reliability; SD-based detection prone to amplification bias
Li W et al. (2018) [102]	T21	ddPCR	Normal = 78, T21 = 28; GA: second trimester	ddPCR with *HLCS* gene and SNP rs6636 differentiates euploid from T21, showing significant ratio differences	Euploid: accuracy = 100%; T21: false negative = 2 cases	Only second trimester samples; unverified BstUI digestion; ethnic variability in SNP rs663
Lee SY et al. (2015) [103]	T21, T18	cdPCR	Low-risk samples = 28, T21 = 10, T18 = 5; GA: 10–35 weeks	cdPCR shows no cross-reactivity in T21 and T18 detection, enabling detection of chromosomal abnormalities at a 1.38% fragment ratio	T21: accuracy = 90%; T18: accuracy = 100%	Cut-off: empirical threshold, not statistically validated; FF: qPCR-based, lacks fetal specificity
Lee SY et al. (2018) [104]	T21	ddPCR	Normal = 827, T21 = 50; GA: 10–22^+3^ weeks	Targeted four sites on Chr21, using size selection to enrich smaller fetal DNA fragments	T21: sensitivity = 100%; overall accuracy = 99.7%	No direct FF measurement
Tan C et al. (2019) [105]	T21, T18	ddPCR	Cut-off value: normal = 30; validation: normal = 26, T21 = 4; GA: 12–25^+2^ weeks	Detected ratios of Chr21/18 with 20 loci probes, using LNA probes for better accuracy	Accuracy = 100%	No T18 validation; no direct FF measurement
Haidong W et al. (2020) [106]	T21, T18, T13	ddPCR	Cut-off value: normal = 50, T21 = 5, T18 = 2, T13 = 1; validation: normal = 201, T21 = 10; GA: 11–27 weeks	The iSAFE NIPT assay detected T21,18,13 in a single ddPCR reaction and can be implemented in decentralized labs, offering a rapid solution within 2.5 h	T21: sensitivity = 100%; specificity = 100%	No T18 or T13 validation samples; no direct FF measurement
Chen X et al. (2021) [107]	T21	ddPCR	Normal = 13, T21 = 2; GA: 14–20 weeks	A computational program was used to design highly specific primers and probes targeting SD on Chr21, for the detection of T21 using ddPCR	T21: accuracy = 100%	ddPCR complexity requires 8 pre-amplifications and 8 reactions; SD limitation: SNPs/CNVs affect amplification
Ramesh M et al. (2023) [108]	Aneuploidies in Chr13,18,21,22,X,Y	ddPCR	NA	A 120-plex assay for aneuploidy detection and a 60-plex assay for fetal fraction quantification successfully detected chromosomal aneuploidies at fetal fractions as low as 4%	Demonstrated strong concordance with NGS, although no specific accuracy rate was provided	NA
Dai P et al. (2022) [73]	T21, T18, T13	ddPCR	Cut-off value: normal = 170; validation: normal = 247, T21 = 25, T18 = 10, T13 = 1; GA: 12–36 weeks	10 sets of primers and probes were used for Chr21,18,13, with dPCR calculating ratios in reference to each other	Sensitivity = 100%; specificity = 95%	High cfDNA input requirement (≥0.2 ng/μL cfDNA); no clear cost analysis
Lassakova S et al. (2023) [109]	T21	ddPCR	Cut-off value: normal = 26, T21 = 16; validation: normal = 24, T21 = 6; GA: 13–18 weeks	16 amplicons from Chr21 and Chr18 (as a reference), with 2 LNA probes to accurately detect reaction products	Sensitivity = 100%; specificity = 100%	Reaction complexity (12/sample): high droplet count (~240 K) for accuracy

Abbreviations: BstUI, Bacillus stearothermophilus U458; cdPCR, chip-based digital polymerase chain reaction; cfDNA, cell-free DNA; Chr, chromosome; CNVs, copy number variations; ddPCR, droplet-based digital polymerase chain reaction; dPCR, digital polymerase chain reaction; FF, fetal fraction; GA, gestational age; HLCS, holocarboxylase synthetase; LNA, locked nucleic acid; mdPCR, microfluidic digital polymerase chain reaction; NA, not available; NIPT, non-invasive prenatal testing; qPCR, quantitative PCR; RCD, relative chromosome dosage; SD, segmental duplication; SNP, single nucleotide polymorphism; T13, trisomy 13; T18, trisomy 18; T21, trisomy 21.

**Table 2:** Summary of dPCR Studies on NIPT for detection of chromosomal microdeletions and microduplications.

Author (Year)	Focus of Study	Method Used	Sample Information	Key Findings/Implications	Diagnostic Accuracy
Wang J et al. (2023) [115]	22q11.2 deletion/duplication syndrome	cdPCR	Normal = 115; duplication = 9; deletion = 6; GA: 17^+1^–27 weeks	Six detection sites in the 22q11.2 region A-D were targeted, using z-scores to differentiate normal from affected samples by comparing copy number ratios	Sensitivity = 73.3%; specificity = 96.5%; PPV = 73.3%; NPV = 96.5%

Abbreviations: cdPCR, chip-based digital polymerase chain reaction; GA, gestational age; NPV, negative predictive value; PPV, positive predictive value.

**Table 3:** Summary of dPCR studies on NIPT or NIPD for monogenetic diseases.

Author (Year)	Focus of Study	Method Used	Sample Information	Key Findings/Implications	Diagnostic Accuracy	Reason/Potential Risk for Low Specificity
Charoenkwan P et al. (2022) [123]	β-thalassemia	cdPCR	35 carriers at risk of having severe β-thalassemia fetuses; GA: 12–18 weeks	The MIB-M/MIB-N ratio was effectively used to differentiate between fetal and maternal DNA	For PIB: sensitivity = 100%; for MIB: sensitivity = 100%; specificity = 92.3%	Maternal DNA interference; low FF; overlapping of MIB-M/MIB-N ratios
Sawakwongpra K et al. (2021) [124]	α and β-thalassemia	ddPCR	46 carriers (22 cases with SEA deletion, 16 cases with HbE, 8 cases with CD41/42 mutation); GA: 17–27 weeks	High accuracy for α-thalassemia; less reliable for β-thalassemia	For SEA deletion: sensitivity = 95.4%, specificity = 91.0%; for HbE: 10 correct, 3 inconclusive, 3 misclassified; for CD41/42 mutation: 2 correct, 4 inconclusive, 2 misclassified	For SEA: low FF (3%) and cfDNA instability in SEA region; for CD41/42 mutation: high ddPCR variability, low positive droplet count, poor probe binding
Suwannakhon N et al. (2023) [125]	β-thalassemia	ddPCR	42 carriers with common mutations (CD41/42, CD17, IVS1-1, CD26); GA: 7–16 weeks	Negative PIB indicates that the fetus is unaffected; positive PIB but negative MIB indicates that the fetus is heterozygous; positive PIB and positive MIB indicates that there is an over-representation of MIBs, and the fetus has compound heterozygous β-thalassemia	100% concordance with those of amniocentesis	Potential false-positive risks: maternal DNA interference; ddPCR allelic imbalance; low cffDNA concentration
D’Aversa E. et al. (2022) [126]	β-thalassemia	ddPCR	52 maternal plasma samples (PIB = 23, β+IVSI-110/N, β039/N; MIB = 30, heterozygous N/M mothers; homozygous β+IVSI-110/β+IVSI-110 fetus = 1); GA: 5–39 weeks	Identified paternally inherited mutations in 23 samples; M/N allelic ratio used to distinguish fetal genotypes for maternally inherited mutations	Classified 51 of 52 samples correctly	M/N ratio at the boundary; limitations of the z-score classification method; statistical method errors
Barrett AN et al. (2012) [127]	Sickle cell anemia	cdPCR	65 maternal plasma samples (45 male and 20 female fetuses); GA: 11^+3^–16^+5^ weeks	The RMD method detected mutations; in female fetuses, indel markers were informative in 65% of cases; allelic ratio analysis distinguished homozygous from heterozygous cases	The classification rate was 82% for male fetuses and 75% for female fetuses; with fetal DNA ≥ 7%, dPCR accuracy = 100%	Delays processing time; indel markers for female fetuses are less effective; long amplicons reduce DNA measurement accuracy; DYS14 copy number differences cause errors
Tsui NB et al. (2011) [128]	Hemophilia A and B	mdPCR	12 samples from 7 hemophilia carriers with male fetuses (hemophilia A = 3, hemophilia B = 4), 20 samples from non-carriers with healthy male fetuses; GA: ≥11 weeks	The RMD method combined with dPCR accurately detected hemophilia A and B in male fetuses as early as 11 weeks of gestation	The fetal genotypes in the 12 plasma samples were detected by dPCR and were found to be consistent with the classifications by the SPRT algorithm	Potential false-positive risks: PCR probe cross-hybridization; cffDNA fraction below 10%; SPRT method’s inability to accurately classify borderline cases
Hudecova I et al. (2017) [129]	Hemophilia A and B	ddPCR	15 carriers of *F8* or *F9* gene variants; GA: 8–42 weeks	Family-specific assays targeted *F8*/*F9* mutations; ZFY/ZFX assays determined fetal sex and DNA; RMD with SPRT classified hemophilia status by allele balance	In 15 pregnancies, 12 were accurately determined, and 3 were unclassified, but no misclassifications occurred	For 3 unclassified: 2 had low FF (0.8%, 4.0%); 1 had much lower total DNA; SPRT failed due to low fetal DNA or few wells
Orhant L et al. (2016) [130]	Achondroplasia	ddPCR	26 samples from women at risk of fetal achondroplasia, 2 samples from normal women and fetuses; GA: third trimester	The combination of ddPCR and mini-sequencing can accurately detect single-point mutations (c.1138G>A and c.1138G>C) in the *FGFR3* gene from maternal plasma	Sensitivity = 100%; specificity = 100% (95% CI, 84.5–100%)	Potential false-positive risks: low cfDNA fragmentation; competition with maternal DNA; low FF
Pacault M et al. (2022) [131]	Achondroplasia, thanatophoric dysplasia (TD), common mutations of the *FGFR3* gene, neurofibromatosis type 1 (NF1), and cystic fibrosis (CF)	ddPCR	202 tests from 175 families at risk for single-gene disorders (achondroplasia = 54, TD = 1, all common mutations of the FGFR3 gene = 4, CF = 69, NF1 = 24); GA: ≥8 weeks	ddPCR detected specific *FGFR3* gene mutations linked to achondroplasia (c.1138G>A, c.1138G>C) and TD (c.742C>T, c.1118A>G, c.1948A>G) from maternal plasma; ddPCR distinguished closely located *CFTR* mutations (c.1519_1521del and c.1521_1523del), overcoming the challenge of genomic proximity; assays for NF1 were designed to distinguish between wild-type and mutant alleles for the identification and quantification of paternal *NF1* mutations	For achondroplasia, TD, and *FGFR3* gene mutation: 19 cases had the *FGFR3* c.1138G>A mutation, 1 had c.1138G>C, and no TD mutations; results fully consistent with invasive prenatal testing and no inconclusive outcomes; for CF: 56% of samples were detected as paternal mutation, 1 case was inconclusive, and results were completely consistent with those of invasive prenatal tests; for NF1: 1 assay for c.2033dup could not be designed	For CF: egg donor’s genetic status was unknown; for NF1: technical limitations caused by a polyC region
Wei X et al. (2020) [78]	SMA (SMN1)	ddPCR	Set A: 17 SMA carriers with male fetuses, Set B: randomly selected 10 women from Set A and analyzed under blinding; GA: 16–22 weeks	The 6th nucleotide of *SMN1* exon 7 was targeted, enabling precise detection of *SMN1* deletions and *SMN1*-to-*SMN2* conversions, both major causes of SMA	The concordance rates with MLPA for Set A and Set B were 94.1% and 90%, respectively, and in all classifiable tests, ddPCR achieved 100% concordance with MLPA	Low FF and concentration
Chang MY et al. (2018) [132]	Hereditary hearing loss	Picodroplet dPCR and cdPCR (used separately)	3 families with known autosomal recessive mutations (*GJB2* c.235delC, *SLC26A4* IVS7-2A>G); GA: 16–27 weeks	Chi-squared and Bayesian analysis predicted fetal genotypes using mutant allele proportions, bypassing the need for fetal DNA fraction or paternal SNPs	Successfully predicted fetal genotypes in all families with high accuracy using both dPCR methods	Potential false-positive risks: borderline mutant allele frequencies; maternal DNA control limitations; inherent dPCR error rates
Gruber A et al. (2018) [133]	NF1 and CF	ddPCR	8 families (NF1 = 4, CF = 4); GA: 8–15 weeks	Identified paternal mutations in *NF1* and *CFTR* mutations	Paternal mutation results were completely consistent with those of invasive prenatal tests	Potential false-positive risks: due to sequence complexity or probe issues; challenges in rare event detection
Debrand E et al. (2015) [134]	CF	ddPCR	1 couple (3 pregnancies) carrying different mutated *CFTR* alleles, 6 normal; GA: 11–12 weeks	Exon 11 of the *CFTR* gene was targeted to quantify the mutant (ΔF508-MUT; FAM) and normal (ΔF508-NOR; VIC) alleles at position c.1521_1523, enabling the detection of paternal *CFTR* mutations	The ΔF508 *CFTR* mutant allele was correctly identified in the three fetuses affected by CF, and it was not detected in the six control fetuses; consistent with traditional invasive testing	Potential false-positive risks: droplet carry-over contamination; low background noise

Abbreviations: cdPCR, chip-based digital polymerase chain reaction; CF, cystic fibrosis; cfDNA, cell-free DNA; cffDNA, cell-free fetal DNA; ddPCR, droplet digital polymerase chain reaction; dPCR, digital polymerase chain reaction; FF, fetal fraction; GA, gestational age; HbE, hemoglobin E; mdPCR, microfluidic digital polymerase chain reaction; MIB, maternally inherited beta-thalassemia allele; MIB-M, MIB mutant allele; MIB-N, MIB wild-type allele; MLPA, multiplex ligation-dependent probe amplification; NF1, neurofibromatosis type 1; PIB, paternally inherited beta-thalassemia allele; RMD, relative mutation dosage; SMA, spinal muscular atrophy; SPRT, sequential probability ratio test; TD, thanatophoric dysplasia.

## Data Availability

Not applicable.
